# Sex as a moderator of the relationship between food addiction and body mass index in young non-help-seeking adults at risk for psychosis and controls

**DOI:** 10.1007/s40519-026-01834-8

**Published:** 2026-03-08

**Authors:** Feten Fekih-Romdhane, Khouloud Akrimi, Nour Bouallègue, Majda Cheour, Souheil Hallit

**Affiliations:** 1 The Tunisian Center of Early Intervention in Psychosis, Department of Psychiatry “Ibn Omrane”, Razi Hospital, 2010 Manouba, Tunisia; 2 Faculty of Medicine of Tunis, Tunis El Manar University, Tunis, Tunisia; 3 School of Medicine and Medical Sciences, Holy Spirit University of Kaslik, P.O. Box 446, Jounieh, Lebanon; 4 Applied Science Research Center, Applied Science Private University, Amman, Jordan

**Keywords:** Sex, Food addiction, Body mass index, Psychosis risk, Young adults, Moderation

## Abstract

**Background:**

Obesity is one of the serious health problems among people with psychosis at the different stages. Therefore, finding effective strategies to mitigate and manage it in a timely manner before complications arise represents a big challenge for the early intervention scientific community. In this study, we aimed to contribute in advancing the field by testing the moderating role of sex between food addiction and body mass index (BMI) in a sample of young people at-risk for psychosis.

**Methods:**

A cross-sectional study was conducted during the period from February to April 2025 among young community adults (aged 18–35) from Tunisia who screened positive to psychosis risk on the Prodromal Questionnaire-Brief (i.e. a total frequency score of 9 and over).

**Results:**

Mean BMI was 24.02 ± 3.83 kg/m2, with 40 (27.0%) of participants in the overweight range, 12 (8.1%) in the obese range. The prevalence of food addiction in our sample was of 16.9%, with a higher prevalence in females (23.0%) relative to males (8.2%). The interaction food addiction by sex (Beta = 0.15; p = 0.041; 95% CI = 0.01; 0.29) was significantly associated with BMI. In females, higher food addiction was significantly associated with higher BMI (Beta = 0.15; p = 0.041; 95% CI = 0.01; 0.29). However, this association was not significant in males.

**Conclusion:**

In light of this study’s results, it is concluded that sex could serve as a significant moderator of the association between food addiction and BMI in community individuals screened as at-risk for psychosis, and should be considered an important determinant in promoting physical health and preventing obesity in this specific group.

*Level of evidence* Level V–Cross-sectional observational study.

## Introduction

There is growing literature documenting that unhealthy physical and lifestyle behaviors precede full-blown psychosis symptoms, that is in those at-risk for psychosis [8]. Accumulating cardiovascular risk factors early in life in this specific group is associated with a 10–25 year reduction in life expectancy among those who will develop the disease [33]. Examples of cardiometabolic risk factors and unhealthy lifestyle behaviors common in people at-risk for psychosis are diets high in saturated fats and calories or low in fiber, protein and nutritional value [9, 30, 39, 50], higher waist circumference relative to age-matched general population [11], and obesity [5]. Recent research looking at anthropometric measurements in individuals at the earliest stage of disease has shown that a high proportion (17.8%) of those at-risk for psychosis are overweight or obese [26]. There is evidence that Body Mass Index (BMI) is in positive correlation with several clinical indicators among at-risk for psychosis population, including polyunsaturated fatty acid levels [2], molecular circulating lipids [32], leptin levels [40], white matter abnormalities [28], positive psychotic symptoms [6], and greater risk of transition to a psychotic disorder [13]. However, to date, anthropometric measures, including BMI, are not well portrayed in at-risk for psychosis individuals [12]. Overweight and obesity are risk factors for developing health problems that are potentially modifiable and preventable. Therefore, the at-risk for psychosis period represents a window opportunity for prevention and early intervention that can also be centered on physical health trajectories in young people aged 18–35 [25]. In this regard, research focused on outcomes such as BMI are crucial to improve understanding of the reasons why young people at-risk for psychosis are at increased risk for becoming obese than those who are not at risk for psychosis and subsequently inform prevention and intervention strategies. To contribute to this line of research, this study proposes to explore food addiction (FA) as a potential correlate of BMI in an at-risk for psychosis group, and to examine sex as a moderating variable.

### The relationship between FA and BMI

Investigating the possible role of FA as a potential root cause of overweight and obesity is attracting growing attention from scholars and practitioners [36]. The concept of FA has gained substantial empirical support from neuroscience and behavioral research, describing food-related brain activities similar to those of substance addiction in the dopaminergic reward system [4, 44], as well as shared personality traits and behavioral deficits, such as high impulsivity levels [60]. For these reasons, and although FA has not been officially introduced as a distinct diagnostic category in international classification systems [3], its assessment has gained importance in recent years. The YFAS 2.0 version developed in 2016 has been the most widely used measure of addictive-like eating behavior that was conceptualized based on the updated criteria for addictive disorders in the DSM-5 [22]. People experiencing FA report problems in resisting eating impulses and have an increased likelihood of consuming immediate disposable high-caloric food [49, 60], and tend to have a higher BMI [51]. There is evidence based on a meta-analysis of 25 studies that FA is highly prevalent (25%) in people with obesity and overweight [51]. The prevalence of FA symptom severity is also high (up to 34,5–38%) in weight loss programs samples [41, 62].

Patients with emerging psychotic disorders reported a higher prevalence of FA (32.1%) compared to both their unaffected siblings (13.0%) and healthy controls (9.0%) [16]. Another study found that 60.6% of people with psychotic disorders were identified as having FA, in whom substantially increased intake of fat, carbohydrate, and high-energy diet was observed [29], which leads in turn to increase in weight and BMI. Research has also shown that at least one in five patients with first episode psychosis (FEP), either unmedicated or newly medicated with antipsychotics, were found to be overweight or have other metabolic syndrome symptoms [42]. Some explanations have been proposed for why people with FA tend to have higher BMI values. For instance, it has been posited that FA might be linked to weight history through comorbid eating disorders, such as Binge-Eating-Disorder [10, 15, 35]. However, this hypothesis contradicted by Pape et al. [47] who demonstrated that FA was significantly associated with higher BMI, even when controlling for Bulimia Nervosa and Binge-Eating-Disorder. In the same study, a higher prevalence of FA was reported in females (18.2%) compared to males (8.6%) [47]. These observations suggest that sex could be a crucial consideration when studying the relationship between FA and BMI.

### Moderating effect of sex

There are numerous possible empirical and theoretical reasons why the moderation effect of sex on the relationship between FA and BMI is expected in this study. There is a large body of literature supporting sex disparity in rates of FA, with a higher mean prevalence among females (12.2%) compared to males (6.4%) [51]. Females were found to exhibit both higher symptom scores and higher prevalence of FA diagnosis [15, 48]. However, these findings could be affected by the samples’ characteristics, as previous studies included predominantly females with overweight/obesity from clinical settings [51]. The sex differences observed in FA result from a joint effect of biological and psychological factors. Females show more susceptibility to develop various forms of addictions due to the influence of hormones [1],World Health Organization, 2020). Besides, females have a higher tendency to turn to food as a way of coping with emotional stressors compared to males [58]. As such, some psychological factors, such as anxiety, have been found to affect subsequent FA only in females and not in males [27]. Furthermore, more females (38.0%) than males (36.9%) in the world have been found to live with overweight or obesity [43]. Moreover, overweight- and obesity-related diseases and increased risk of mortality occur at greater frequency and at lower BMIs in females than males (J. [23]). Taken together, these data suggest that sex differences in the relationship between FA and BMI are plausible. However, no studies could be found that examined how the link between addictive-like eating behavior and weight may vary across sex, and this research question is yet to be addressed.

### Rationale and Objectives

Despite its major clinical significance, physical health in young people at-risk for psychosis remains largely under-researched [21], while more attention was devoted to other aspects, such as substance use. Investigating this specific group offers the opportunity to advance our understanding of how to improve physical health trajectories since the very early stages of the disease [25]. For this reason, growing international calls have been made for early physical health intervention for young people with psychosis [55]. The current study has as main goal to examine the moderating role of sex between FA and BMI in a group of non-help-seeking young adults at-risk for psychosis. Guided by earlier evidence, it is anticipated that there will be a positive correlation between FA and BMI, and that this correlation will be stronger for females as compared to males.

## Methods

### Design, procedure and study sample

A cross-sectional study was conducted during the period from February to April 2025. The recruitment procedure was completed using the snowball sampling method. Potential participants were recruited through different social media sites (Instagram, WhatsApp, Facebook, Telegram, etc.) and online forums. Members of research team contacted potential participants to invite them to take part in the study. Then, they were asked to assist researchers in identifying other persons who would be interested to participate through their personal, social or professional networks. Inclusion and exclusion criteria are presented in Table 1.

**Table 1:** Inclusion and exclusion criteria

Inclusion criteria	Exclusion criteria
(1) Having an age comprised between 18 and 35, the range to which predominantly belongs the at-risk for psychosis population [14] (2) Being able to read and understand the Arabic language (3) Being from Tunisia and residing in the country at the time of the survey (4) Not having a self-reported diagnosed psychotic disorder or prior antipsychotics intake (6) Having access to the Internet	(1) Not meeting the inclusion criteria (2) Not consenting to participate

A link to the questionnaire was created using Google Forms. The questionnaire was available only in the Arabic language and took around 10 min to complete. No compensation was offered for participation. All necessary information about the study was provided to participants. Confidentiality of personal data and anonymity of subjects were preserved. A Yes/No consent statement was added at the beginning of the survey. The study protocol was approved by the ethics committee of Razi hospital, Manouba, Tunisia (Ref: ECRPH-2025-0022; Date: 25/02/2025). A total of 444 responses were received through the link, among whom 13 answered “no” to the consent question and were not allowed to complete the survey, leaving a total final sample of 431 eligible participants.

### Minimum sample size

The G-power software v.3.0.10 estimated a minimum sample of 335 participants, using the multiple regression option, based on an R^2^ of 0.05, an alpha error of 5%, a power of 80% and 10 predictors to be entered in the moderation analysis.

### Measures

#### Sociodemographic data

Participants were requested to provide information related to their demographic characteristics including sex, age, marital status, educational attainment, residency, tobacco use, alcohol use, lifetime cannabis use, and personal history of any previously diagnosed psychiatric illness other than psychosis.

#### BMI

BMI was calculated by dividing weight in kg by height in meters squared, with participants characterized according to the following World Health Organization cut-off values: obese ≥ 30, overweight ≥ 25, normal 18.50 – 24.99, and underweight < 18.5 (World Health Organization, 1995).

#### The prodromal questionnaire- brief (PQ-B)

The PQ-B was used to screen for at-risk for psychosis status of individuals from the general population. This is a self-administered instrument that contains 21 dichotomous no/yes items [37, 38]. A total “frequency” score can be calculated by summing up the items’ scores, and varies between 0 and 21. For each item answered “yes”, a score of 1 point is received and the respondent is asked to further score the subsequent related distress on a scale from 1 (strongly disagree) to 5 (strongly agree). Only the PQ-B frequency score was considered in the present study, and surpassing a cut-off of 9 or higher allowed to define those at-risk for psychosis [17, 54]. The Arabic validated version of the PQ-B was used [17], which yielded a Cronbach’s α of 0.90 in our sample.

### The modified version of the yale food addiction scale (mYFAS 2.0)

The mYFAS 2.0 was used to assess the severity of self-reported FA symptoms in our sample [34]. The scale consists of a self-administered tool of nine items: seven of them reflect the different DSM criteria for substance use disorders, while the two others evaluate clinical significance of symptoms. Each item is rated on a five-point scale from 1 (never) to 5 (4 or more times a week or daily). A FA status was defined as having at least 3 of the 7 symptoms and meeting the criterion for clinical significance (Alan J [19, 20, 34]). The Arabic validated version of the mYFAS 2.0 was adopted in our study [24], yielding a Cronbach alpha value of 0.85.

### Statistical analysis

To prevent duplication of responses, all datasets were screened in Microsoft Excel. No duplicates were identified, ensuring that each participant contributed a single unique entry in the final dataset. The SPSS v.27 software was used for the statistical analysis. The BMI score was considered normally distributed since the skewness and kurtosis values were between the -1; + 1 interval. The Student t-test was used to compare a continuous variable and a dichotomous variable one-way ANOVA to compare a continuous variable and a categorical variable, and Pearson's test to correlate two continuous variables. The moderation analysis was performed using PROCESS MACRO (a SPSS add-on) v3.4 Model 1. Interaction terms were probed by examining the association of food addiction with BMI between males and females. Covariates entered in the model were those that showed a p < 0.25 in the bivariate analysis. P < 0.05 was considered statistically significant.

## Results

### Participants

Table 2 contains a comparison between participants with no risk vs at-risk of psychosis. From those 431 persons, 148 participants screened positive for an ultra-high risk of psychosis (PQ-B scores ≥ 9), with a mean age of 23.84 years and 58.8% females (Table 2). In the at-risk group, the mean BMI was 24.02 ± 3.83 kg/m2 (23.84 ± 4.05 among females and 24.29 ± 3.49 among males), with 40 (27.0%) of participants in the overweight range, 12 (8.1%) in the obese range, and 96 (64.9%) having a healthy BMI. The proportion of individuals with FA status was higher among participants at-risk for psychosis (16.9%) compared to those non-at-risk (12.4%). In those at-risk for psychosis, a higher prevalence of FA was found in females (23.0%) relative to males (8.2%). The most common FA symptoms endorsed were continued use despite knowledge of adverse consequences (79.7%) and tolerance (73.0%) (Table 3).

**Table 2:** Sociodemographic and other characteristics of the sample (N = 431)

Variable	N (%)	Participants who screened negative for an ultra-high risk of psychosis (PQ-B scores < 9) (n = 282; 65.4%)	Participants who screened positive for an ultra-high risk of psychosis (PQ-B scores ≥ 9) (n = 149; 34.6%)	*p*
Sex				0.812
Male	176 (40.8%)	114 (64.8%)	62 (35.2%)	
Female	255 (59.2%)	168 (65.9%)	87 (34.1%)	
Marital status				0.567
Single, divorced	407 (94.4%)	265 (65.1%)	142 (34.9%)	
Married	24 (5.6%)	17 (70.8%)	7 (29.2%)	
Educational attainment				**0.015**
High-school level	8 (1.9%)	2 (25.0%)	6 (75.0%)	
University level	423 (98.1%)	280 (66.2%)	143 (33.8%)	
Residency				0.495
With family	258 (59.9%)	174 (67.4%)	84 (32.6%)	
With friends	105 (24.4%)	64 (61.0%)	41 (39.0%)	
Alone	68 (15.8%)	44 (64.7%)	24 (35.3%)	
Personal history of mental health problems other than psychotic disorders				** < 0.001**
No	343 (79.6%)	239 (69.7%)	104 (30.3%)	
Yes	88 (20.4%)	43 (48.9%)	45 (51.1%)	
Tobacco use				**0.022**
No	326 (75.6%)	223 (68.4%)	103 (31.6%)	
Yes	105 (24.4%)	59 (56.2%)	46 (43.8%)	
Alcohol use				0.550
No	334 (77.5%)	221 (66.2%)	113 (33.8%)	
Yes	97 (22.5%)	61 (62.9%)	36 (37.1%)	
Lifetime cannabis use				0.276
No	323 (74.9%)	216 (66.9%)	107 (33.1%)	
Yes	108 (25.1%)	66 (61.1%)	42 (38.9%)	
	**Mean ± SD**	**Mean ± SD**	**Mean ± SD**	
Age (years)	24.41 ± 4.51	24.57 ± 4.36	24.11 ± 4.79	0.322
Body Mass Index (kg/m^2^)	23.89 ± 3.56	23.79 ± 3.38	24.08 ± 3.88	0.418

**Table 3:** Endorsement of each symptom of the mYFAS 2.0 and food addiction scores among participants

	Total sample	Participants who screened negative for an ultra-high risk of psychosis (PQ-B scores < 9)	Participants who screened positive for an ultra-high risk of psychosis (PQ-B scores ≥ 9)
**Symptom**	**N (%)**	**N (%)**	**N (%)**
1. Food taken in larger amount and for longer period than intended	43 (10.0%)	25 (8.9%)	18 (12.2%)
2. Persistent desire or repeated unsuccessful attempt to quit certain foods	27 (6.3%)	20 (7.1%)	7 (4.7%)
3. Much time/activity to obtain, use, recover	76 (17.6%)	42 (14.9%)	34 (23.0%)
4. Important occupational, social, or recreational activities reduced or given up	43 (10.0%)	26 (9.2%)	17 (11.5%)
5. Characteristic withdrawal symptoms when cutting down on certain foods; substance taken to relieve withdrawal	47 (10.9%)	19 (6.7%)	28 (18.9%)
6. Consumption of the same types or amounts of food continues despite knowledge of adverse consequences	345 (80.0%)	226 (80.1%)	118 (79.7%)
7. Tolerance (marked increase in amount; marked decrease in effect)	330 (76.6%)	221 (78.4%)	108 (73.0%)
8. Significant distress	58 (13.5%)	31 (11.0%)	27 (18.2%)
9. Impaired functioning	67 (15.5%)	37 (13.1%)	30 (20.3%)
**Food addiction Total scores** by using the mYFAS, Mean ± SD	9.48 ± 7.92	8.41 ± 7.76	11.56 ± 7.84
**Food addiction status** (% yes) ^a^			
Total sample	60 (13.9%)	35 (12.4%)	25 (16.9%)
Males	8 (4.5%)	3 (2.6%)	5 (8.2%)
Females	52 (20.4%)	32 (19.0%)	20 (23.0%)

^*a*^* Food addiction status was based on presenting at least 3 of the 7 dependence symptoms and meets the criterion for clinical significance (A. J. *[19, 20]*)*

## Bivariate analysis of factors associated with BMI score

Males vs females, married participants, those living alone and having a personal history of mental health problems were significantly associated with higher body mass index in the total sample and in the group with no risk of psychosis (Table 4).

**Table 4:** Bivariate analysis of factors associated with body mass index

	Total sample			Participants who screened negative for an ultra-high risk of psychosis (PQ-B scores < 9)			Participants who screened positive for an ultra-high risk of psychosis (PQ-B scores ≥ 9)		
**Variable**	Mean ± SD	***p***	**Effect size**	Mean ± SD	***p***	Effect size	Mean ± **SD**	***p***	Effect size
Sex		**0.011**	0.251		**0.011**	0.311		0.479	0.118
Male	24.42 ± 3.34			24.41 ± 3.19			24.29 ± 3.49		
Female	23.53 ± 3.66			23.37 ± 3.45			23.84 ± 4.05		
Marital status		** < 0.001**	0.697		**0.040**	0.515		**0.030**	0.912
Single, divorced	23.76 ± 3.50			23.69 ± 3.35			23.88 ± 3.77		
Married	26.21 ± 3.82			25.42 ± 3.46			27.33 ± 4.01		
Educational attainment		0.292	0.377		0.448	0.539		0.109	0.673
High-school level	22.58 ± 2.48			25.60 ± 0.19			21.57 ± 1.93		
University level	23.92 ± 3.57			23.78 ± 3.39			24.13 ± 3.85		
Residency		**0.013**	0.020		** < 0.001**	0.054		0.818	0.003
With family	23.55 ± 3.32			23.23 ± 2.94			24.11 ± 3.84		
With friends	24.03 ± 4.02			24.23 ± 3.96			23.71 ± 4.15		
Alone	24.96 ± 3.49			25.34 ± 3.57			24.26 ± 3.29		
Personal history in mental health problems		** < 0.001**	0.616		**0.009**	0.435		** < 0.001**	0.765
No	23.46 ± 3.35			23.57 ± 3.28			23.20 ± 3.49		
Yes	25.59 ± 3.87			25.03 ± 3.69			25.97 ± 3.91		
Tobacco use		0.526	0.071		0.363	0.134		0.962	0.008
No	23.83 ± 3.54			23.70 ± 3.37			24.03 ± 3.80		
Yes	24.08 ± 3.63			24.15 ± 3.43			24.00 ± 3.92		
Alcohol use		0.389	0.099		0.565	0.083		0.469	0.139
No	23.81 ± 3.56			23.73 ± 3.36			23.89 ± 3.86		
Yes	24.17 ± 3.55			24.01 ± 3.47			24.43 ± 3.72		
Lifetime cannabis use		0.203	0.129		0.160	0.198		0.804	0.040
No	23.78 ± 3.70			23.63 ± 3.44			23.98 ± 4.12		
Yes	24.24 ± 3.07			24.30 ± 3.14			24.13 ± 3.00		

Numbers in bold indicate significant p values.

Married participants and having a personal history of mental health problems were significantly associated with higher body mass index in the group at-risk of psychosis.

Moreover, older age (r = 0.18; p < 0.001) and higher food addiction (r = 0.38; p < 0.001) was significantly associated with higher BMI in the total sample and in participants at risk of psychosis. Moreover, in the group with no risk of psychosis, higher food addiction (r = 0.39; p < 0.001), but not age (r = 0.11; p = 0.076), was significantly associated with higher BMI.

### Analysis of moderation

The interaction FA by sex (Beta = 0.15; p = 0.041; 95% CI 0.01; 0.29) was significantly associated with BMI, R^2^ = 0.376, F = 10.47, Intercept Beta = 19.10, p < 0.001 (Table 5, Model 1). In females, higher FA was significantly associated with higher BMI (Beta = 0.15; p = 0.041; 95% CI 0.01; 0.29). However, this association was not significant in males (Table 4).

**Table 5:** Moderation analysis taking food addiction as the independent variable, sex as the moderator and body mass index as the dependent variable

	Beta	***t***	***p***	95% CI
**Model 1: total sample (F = 10.49; R**^**2**^** = 0.183; intercept Beta = 23.38; p < 0.001)**				
Food addiction	− 5.09	− 2.10	**0.036**	− 9.86; − 0.33
Gender	− 1.35	− 3.87	** < 0.001**	− 2.04; − 0.66
Interaction of food addiction by gender	4.01	3.09	**0.002**	1.46; 6.56
**Model 2: participants who screened negative for an ultra-high risk of psychosis (PQB scores < 9) (F = 5.63; R**^**2**^** = 0.157; intercept Beta = 24.63; p < 0.001)**				
Food addiction	− 4.04	− 1.05	0.293	− 11.58; 3.51
Gender	− 1.31	− 3.17	**0.002**	− 2.12; − 0.50
Interaction of food addiction by gender	3.21	1.60	0.111	− 0.74; 7.16
**Model 3: participants who screened positive for an ultra-high risk of psychosis (PQB scores ≥ 9) (F = 7.11; R**^**2**^** = 0.315; intercept Beta = 21.90; p < 0.001)**				
Food addiction	− 6.26	− 1.93	0.055	− 12.67; 0.15
Gender	− 1.53	− 2.43	**0.016**	− 2.78; − 0.29
Interaction of food addiction by gender	5.02	2.81	**0.006**	1.48; 8.55

Numbers in bold indicate significant p values.

The interaction FA by sex (Beta = 3.12; p = 0.125; 95% CI -0.87; 7.11) was not significantly associated with BMI in participants who screened negative for an ultra-high risk of psychosis, R2 = 0.118, F = 4.58, Intercept Beta = 27.08, p < 0.001 (Table 4, Model 2). However, the interaction was significant in participants who screened positive for an ultra-high risk of psychosis (Beta = 4.09; p = 0.022; 95% CI 0.59; 7.59), R2 = 0.340, F = 9.03, Intercept Beta = 18.66, p < 0.001 (Table 4, Model 3). In females who screened positive for prodromal symptoms, higher FA was significantly associated with higher BMI (Beta = 3.62; p < 0.001; 95% CI 1.91; 5.33). However, this association was not significant in males (Table 6, Model 2).

**Table 6:** Conditional effect of the focal predictor (food addiction) at values of the moderator (sex)

	Beta	***t***	***p***	95% CI
**Model 1: total sample**				
Male	-1.08	-0.91	0.363	-3.41; 1.25
Female	2.94	5.39	** < 0.001**	1.86; 4.01
**Model 2: participants who screened positive for an ultra-high risk of psychosis (PQB scores ≥ 9)**				
Male	-1.25	-0.80	0.428	-4.34; 1.85
Female	3.77	4.24	** < 0.001**	2.01; 5.53

Numbers in bold indicate significant p values.

## Discussion

Obesity is one of the serious health problems among people with psychosis at the different stages. Therefore, finding effective strategies to mitigate and manage it in a timely manner before complications arise represents a big challenge for the early intervention scientific community. In this study, we aimed to contribute in advancing the field by testing the moderating role of sex between FA and BMI in a sample of young people at-risk for psychosis as defined by a PQ-B cut‐off score of ≥ 9 points in frequency versus a control group identified as non-at-risk for psychosis (PQ-B < 9). Findings showed that those at-risk for psychosis had higher scores of FA and greater BMI. Our hypothesis was partially supported, with the relationship FA-BMI being significant only in females who screened positive for an at-risk for psychosis state.

The prevalence of FA in those at-risk for psychosis was of 16.9% (23.0% in females vs. 8.2% in males), which is in line with the limited existing body of literature. A previous study conducted in Tunisia showed that unaffected siblings of patients with schizophrenia, who are considered part of the high-risk group due to their genetic predisposition, had a significantly higher prevalence of FA compared to healthy controls from the general population (13% vs 9%, respectively) [16]. In addition, we found a mean BMI of 24.02 ± 3.83 kg/m^2^ in the at-risk for psychosis group, with 35.1% being classified as overweight or obese. Consistent with our results, a recent study by Husain et al. [26] found a mean BMI of 21.42 ± 4.11 kg/m2 in individuals at risk for psychosis from Pakistan, with 17.8% being overweight or obese. Teasdale et al. [57] found a prevalence of FA of 50% and a BMI of 24.7 ± 3.9 kg/m^2^ in a small sample of 10 people at ultra-high risk for psychosis recruited from the youth mental health services in Australia, which were significantly higher to those observed in the group of participants diagnosed with depression or anxiety (FA 10% and BMI 21.8 ± 1.1 kg/m2). As a potential explanation to these patterns, Romer et al. (A. L. [52, 53]) demonstrated that dopamine genetic risk is linked to greater FA symptoms, and in turn to higher BMI, via decreased reward-related ventral striatum activity. This biological pathway considered as explaining factor of propensity for FA and, ultimately, likelihood for gaining weight, might also explain why people at-risk for psychosis, who have well-established dopamine alterations [59], may develop both FA and obesity.

Another relevant result of our research is that correlations of FA to BMI in young people at-risk for psychosis differed across sex, being only significant for females. Little or no information is available so far on whether any differences on the link between FA and BMI across sex exist and could be informative for prevention and intervention efforts. Existing evidence indicates that FA symptoms and status are overrepresented in females compared to males [51], which can likely be explained by a combination of hormonal [1], emotional [58] and psychological [27] factors. Throught the life developmental stages, females experience more loss-of-control eating than males, with sex disparities being more pronounced in late adolescence and young adulthood [18, 56]. Differences between males and females were also seen in nature, intensity, and frequency of food cravings, as well as subsequent negative emotions [23, 31, 46, 61]. On the other hand, previous literature indicated that overweight/obesity and their related consequences are documented more frequently among females [23, 43]. Nevertheless, the vast majory of research to date that has directly tested the effect of sex on FA and BMI has been conducted in clinical settings among sex-unbalanced samples. Given the major clinical relevance and potential implications of moderation effect of sex between addictive-like eating behavior and weight, further investigations of these interactions across the psychosis spectrum are warranted.

### Study limitations

Our study did not collect data at different time points or report follow-up results. Therefore, the findings are only correlational in nature and do not allow any causal conclusions regarding the moderation mechanisms affecting the associations between FA and BMI. Additionally, the use of self-reported instrument to screen for psychosis risk might have led to recall bias. Moreover, the adoption of an online recruitment method and a snowball technique can limit the generalizability of findings. Indeed, this sampling strategy can lead to potential bias by recruiting a highly health-aware or digitally-active population. Finally, our findings should be interpreted with caution because a number of potential confounding factors, such as physical activity or dietary preferences and composition, were not controlled for in this study. A limitation of this study is the use of BMI, which does not differentiate between body fat mass and body lean mass [45]. In other terms, BMI is connected to but is not a direct measure of body fat. Therefore, it is useful in screening for obesity, but it does not displace clinical judgment and cannot be a diagnostic measure of obesity.

### Potential clinical and research implications

Despite evidence highlighting the beneficial effects of promoting physical health and lifestyle behaviors in people at-risk for psychosis, physical health determinants are often neglected and not routinely monitored even in specialized early intervention services [7]. Our findings and previous literature suggest that FA is more prevalent and mean BMI values tend to be higher in at-risk for psychosis groups compared to control subjects. The reasons for this are still unknown and can be varied, either due to the psychotic symptoms themselves or to other emotional and social reasons. As speculated in previous research (Adrienne L [52, 53]), our results lend further support to the utility of FA in obesity prevention strategies, policies and research. The current evidence provided for the moderating effect of sex on the relationship between FA and BMI in the at-risk group supports the development of specific interventions targeted to change addictive eating behaviors in the at-risk population, particularly in females. In other words, measures to promote health and manage or prevent obesity in at-risk for psychosis people should be customized to sex for more effectiveness. Regarding future research directions, researchers are called to replicate this study’s model in different contexts such as other countries, cultures, and clinical settings (e.g., UHR or FEP subjects), and using a prospective design, in order to confirm or refute our results and reveal possible mechanisms by which FA can be related to BMI along the psychosis continuum.

## Conclusion

In light of this study’s results, it is concluded that sex could serve as a significant moderator of the association between FA and BMI in community individuals screened as at-risk for psychosis, and should be considered an important determinant in promoting physical health and preventing obesity in this specific group. This area of research might benefit from additional experimental studies testing whether targeting FA could decrease the incidence of obesity in people at early stages of psychosis.

## Data Availability

The datasets generated and/or analyzed during the current study are publicly available: https://osf.io/sqw7m/
